# Complement factor H binding by different Lyme disease and relapsing fever *Borrelia *in animals and human

**DOI:** 10.1186/1756-0500-2-134

**Published:** 2009-07-15

**Authors:** Mangesh R Bhide, Raquel Escudero, Emilio Camafeita, Horacio Gil, Isabel Jado, Pedro Anda

**Affiliations:** 1 Laboratorio de Espiroquetas y Patógenos Especiales, Centro Nacional de Microbiología, Instituto de Salud Carlos III, Majadahonda, Madrid, Spain; 2 Unidad de Proteómica, Centro Nacional de Investigaciones Cardiovasculares, Instituto de Salud Carlos III, Madrid, Spain; 3 Laboratory of Biomedical Microbiology and Immunology, University of Veterinary Medicine, Kosice, Slovakia; 4 Neuroimmunological Institute, Slovak Academy of Sciences (SAS), Bratislava, Slovakia

## Abstract

**Background:**

*Borreliae *employ multiple immune evasive strategies such as binding to complement regulatory proteins [factor H (fH) and factor H like-1 (FHL1)], differential regulation of surface membrane proteins, antigenic variation, and binding of plasminogen/plasmin and matrix metalloproteinases. As a complement regulatory subunit, fH serves as a cofactor for the factor I-mediated cleavage of C3b. fH binding by *Borrelia *has been correlated with pathogenesis as well as with host diversity. Here we show the differential binding of borrelial proteins to fH from human and animal sera.

**Findings:**

Affinity ligand binding experiments, 2-D electrophoresis, and protein identification and peptide *de novo *sequencing based on mass spectrometry, revealed novel fH putative binding proteins of Lyme- and relapsing fever *Borrelia*. An OspA serotype-associated differential human and animal fH binding by *B. garinii *was also observed, which could be related with the ability of some strains from serotypes 4 and 7 to invade non-nervous system tissues. Also, the variable affinity of binding proteins expressed by different *Borrelia *to animal fH correlated with their host selectivity.

**Conclusion:**

The novel animal and human putative fH binding proteins (FHBPs) in this study underscore the importance of evasion of complement in the pathogenesis of *Borrelia *infections.

## Findings

Binding of fH on the borrelial cell surface is critical for resistance to complement-mediated killing by inhibiting the formation of the terminal complement complex [1,2]. Human fH binding has been reported and its association with the pathogenic nature of *Borrelia *species was predicted earlier [1,3,4]. Complement resistant strains (e.g. *B. afzelii *and *B. hermsii*) survive successfully in body compartments where complement concentration is high, whereas it is proposed that *B. garinii *strains do not bind fH on their surface and thus are prone to complement-mediated killing; therefore, they would be able to invade the nervous system where complement concentration is low [5]. However, it has been reported that some OspA serotypes of *B. garinii *can infect and disseminate through the skin [6], and resist human complement mediated killing [7].

The nature of human and animal fH binding ability to *Borrelia *is complex. To date, the majority of studies have focused on human fHBPs of *B. burgdorferi *s.s., *B. afzelii *and *B. hermsii*, using purified fH or recombinant proteins [3,8-10]. In contrast, here we have analyzed a wider panel of *Borrelia *species, as well as human and different animal sera as source of native fH. We also present how the reservoir competence for *Borrelia *parallels their fH binding ability in different animal species, identifying known as well as not yet described putative fHBPs.

## Materials and methods

**Figure 1:**
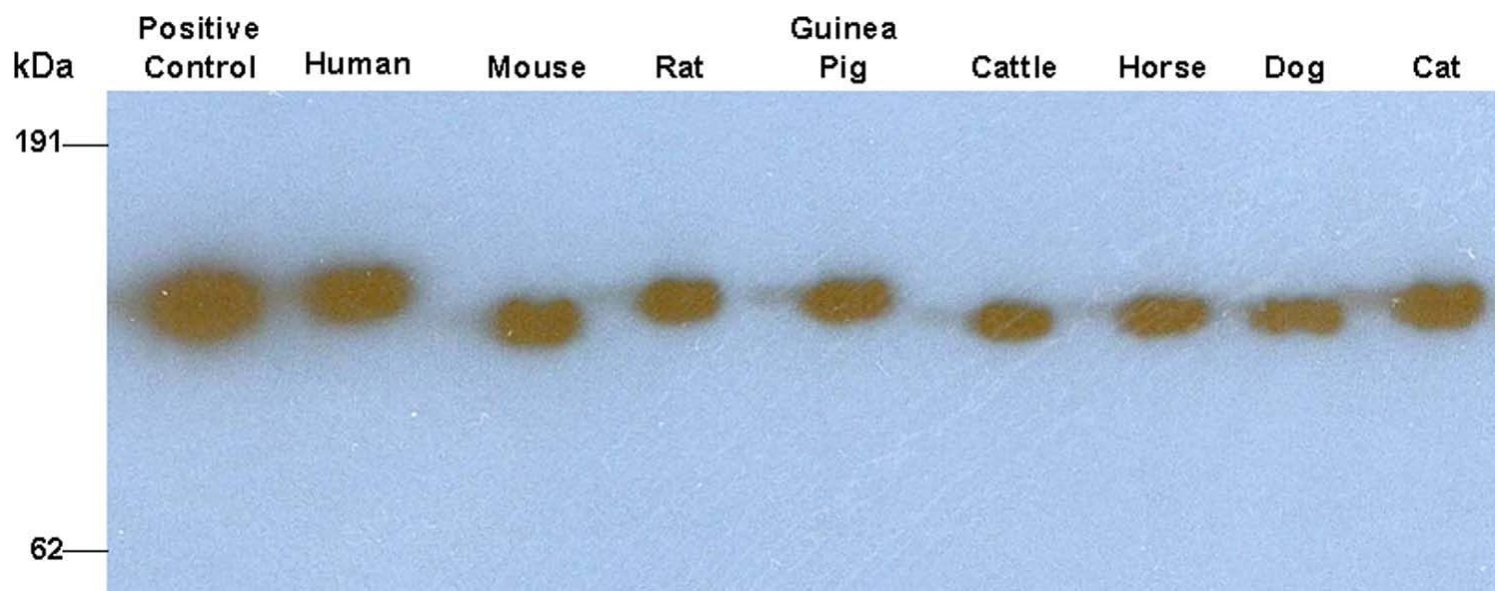
**Binding ability of anti-factor H antibody to human and animal fH**. Non-reducing one-dimensional immunoblot of albumin depleted human and animal sera against sheep anti-human fH polyclonal antibody. Purified human fH was used as a positive control.

First, the reactivity of sheep anti-human fH polyclonal antibody to fH from human and different animal species was assessed (Figure 1). Human and animal (mouse, rat, guinea pig, cattle, horse, dog and cat) serum samples, free of antibodies against *B. burgdorferi*, were purchased (Sigma-Aldrich), albumin depleted [11]., fractionated by non-reducing SDS-PAGE (10 μg/well/animal species) and transferred to nitrocellulose membranes. Purified human fH served as a positive control. Membranes were blocked overnight at 4°C in SuperBlock buffer (Pierce, Rockford, IL, USA) and then incubated for 2 hours (37°C with shaking) with sheep anti-human fH polyclonal antibody (Abcam, Cambridge, UK) diluted 1:1,500 in TTBS buffer [10 mM Tris/HCl (pH 8.3), 0.05% Tween-20 and 150 mM NaCl] with 1% skim milk. Membranes were washed 3 times with TTBS, incubated with rabbit anti-sheep HRPO antibody (Abcam) diluted to 1:400,000 in TTBS with 1% skim milk for 1 hour (37°C with shaking) and then washed 3 times as above. The reaction was developed by chemiluminescence with SuperSignal West Dura substrate (Pierce).

Subsequently, affinity ligand binding immunoblot (ALBI) assays were performed to detect fHBPs of Lyme disease and relapsing fever borreliae (Table 1). Borrelial strains were grown in BSK-II medium at 33°C, harvested, washed 5 times with PBS supplemented with 5 mM MgCL_2 _and then resuspended in ultra pure water containing 1% trifluoroacetic acid (Sigma-Aldrich), 1% of nuclease mix and 1% of a protease inhibitor cocktail (GE Healthcare). Cells were sonicated and total protein concentration was measured (Bradford). Proteins were fractionated by non reducing SDS-PAGE, immunoblotted, and the membranes were cut in 3 mm strips, which were incubated 2 hours either with 1 ml of purified human fH (1,500 μg/ml) as a positive control or human and animal sera (1:4 dilution). fH bound to borrelial proteins was detected with sheep anti-human fH antibody and rabbit anti-sheep HRPO conjugate, as indicated above. Binding was detected by chemiluminescence.

**Table 1:** fH binding proteins, binding strength, calculated molecular mass and isoelectric points estimated in 2-DE.

***Borrelia *species (strain)**	**OspA Serotype**	**MW of fHBP**	**Estimated pI^*a*^**	**Human and animal fH binding affinity**							
				
				**H**^*b*^	**M**	**R**	**G**	**C**	**Ho**	**D**	**Ca**
*B. burgdorferi *s.s. (SKT2)	1	**~26 kDa**^*c*^	8.0-8.1	+++^*d*^	+++	-	-	-	-	-	-

*B. afzelii *(SKT4)	2	**~15 kDa**	4.0-5.2	-	+++	-	-	-	-	+++	++
		**~26 kDa**	6.8-7.1	+++	++	-	-	-	-	-	-

*B. garinii *(Rio2)	3	-	-	-	-	-	-	-	-	-	-

*B. garinii *(PBi)	4	**~19 kDa**	6.0-7.0	+++	-	-	-	-	-	-	-
		**~28 kDa**	5.0-5.3	-	+++	-	-	-	-	-	-

*B. garinii *(G117)	5	~26 kDa	5.6-6.2	-^*e*^	++	-	-	-	-	-	-

*B. garinii *(SKT3)	6	-	-	-	-	-	-	-	-	-	-

*B. garinii *(T25)	7	~17 kDa	5.0-5.5	++	-	-	-	-	-	-	-

*B. garinii *(CL1)	8	-	-	-	-	-	-	-	-	-	-

*B. valaisiana *(VS116)	NA^*f*^	~17 kDa	4.2-5.0	+++	-	-	-	-	-	+++	-

*B. andersonii *(21123)	NA	~15 kDa	5.5-6.1	+	-	-	-	-	-	-	-
		~17 kDa	8.0-8.8	+	-	-	-	-	-	+	-
		~23 kDa	6.1-6.6	+	++	-	-	-	-	-	-
		~26 kDa	8.0-8.3	-	++	-	-	-	-	-	-

*B. lusitaniae *(Poti B2)	NA	-	-	-	-	-	-	-	-	-	-

*B. bissettiii *(DN127)	NA	~25 kDa	8.0-8.5	+	-	-	-	-	-	-	-
		~28 kDa	5.0-5.5	-	+++	-	-	-	-	-	-
		~40 kDa	6.9-7.8	-	+	-	-	-	-	-	-

*B. japonica *(HO14)	NA	~15 kDa	4.8-5.2	-	+++	++	-	-	-	-	-
		**~19 kDa**	4.5-5.0	-	-	++	-	-	-	-	-
		**~22 kDa**	6.0-6.8	+	-	-	-	-	-	++	++
		~24 kDa	4.5-4.9	-	-	++	-	-	-	-	-
		~26 kDa	7.9-8.3	-	+++	-	-	-	-	-	-

*B. hermsii *(HS1)	NA	**~20 kDa**	8.0-8.3	+++	+++	+++	+++	-	-	-	-

*B. parkeri *(M3001)	NA	~23 kDa	8.0-8.5	++	-	-	-	-	-	-	-

*B. anserina *(ES-1)	NA	-	-	-	-	-	-	-	-	-	-

*B. coriaceae *(Co53)	NA	~40 kDa ~**58 kDa**	7.0-8.0 5.5-5.7	- -	+++ +++	- ++	- -	+++ +++	- -	- -	- -

^*a *^pI: isoelectric point; ^*b *^H: human; M: mouse; R: rat; G: guinea pig; C: cattle; Ho: horse; D: dog; Ca: cat; ^*c *^boldface indicate those proteins identified by the mass spectrophotometry and related analysis in this study;^*d *^fH binding strength. +++: high; ++: medium; +: weak; -: negative; ^*e *^This protein was positive when purified human fH was used; ^*f *^NA: not applicable;

To isolate and identify the borrelial proteins showing human/animal fH binding ability, 2-D electrophoresis (2-DE) coupled with MALDI-TOF-TOF was employed. For that, borrelial proteins were cleaned (Bio-Rad Laboratories S.A., Barcelona, Spain) and solubilized in Destreak solution (GE Healthcare, Madrid, Spain). Protein solutions (100 μg) were loaded by rehydration on 7 cm IPG strips (pH 3-11NL or pH 4-7; GE Healthcare), and were focused for 9,142 Vhr using the IPGphor system (GE Healthcare). Strips were equilibrated and subjected to SDS-PAGE on duplicated 15% polyacrylamide gels. One gel was stained with the Silver Stain Plus Kit (Bio-Rad), and the second was subjected to ALBI assay as described above, to ascertain fHBPs. The protein spots of interest were excised from 2-DE gels and digested [12] (Proteineer, Bruker-Daltonics). Digested aliquots were mixed with α-cyano-4-hydroxycinnamic acid in 33% aqueous acetonitrile and 0.25% trifluoroacetic acid. This mixture was deposited onto a 600 μm AnchorChip prestructured MALDI probe (Bruker-Daltonics) and allowed to dry. MALDI-MS data were obtained in an automated analysis loop (Ultraflex, Bruker-Daltonics) equipped with a LIFT-MS/MS device. Spectra were acquired in the positive-ion mode at 50 Hz laser frequency, and 100 to 1000 individual spectra were averaged. Automated analysis of mass data was performed (FlexAnalysis software; Bruker-Daltonics). MALDI-MS and MALDI-MS/MS data were combined (BioTools, Bruker-Daltonics) to search a non-redundant protein database (NCBInr) using Mascot software (Matrix Science). When Mascot search failed to assign a peptide match with *Borrelia *proteins, manual *de novo *sequencing [13] was attempted based on MALDI-MS/MS spectra.

As coiled-coil elements were demonstrated to be involved in the presentation of the fH binding sites [14,15]., putative coiled-coil formation analysis for novel sequences was performed by using PepCoil software [16]. Lipoprotein signal peptide analysis was done following the description of Setubal *et al*. [17].

## Results

### A wide repertoire of human fHBP was detected in Borrelia species

**Figure 2:**
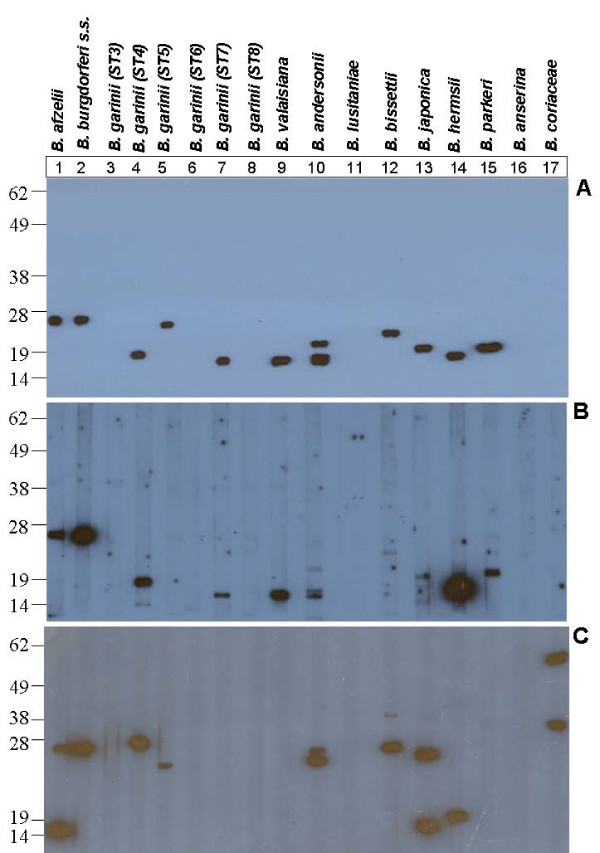
**Affinity ligand binding (ALBI) assays**. Non-reducing one-dimensional immunoblot of whole cell sonicates of different *Borrelia *species against purified human fH (Panel A), human serum (Panel B), and mouse serum (Panel C).

**Figure 3:**
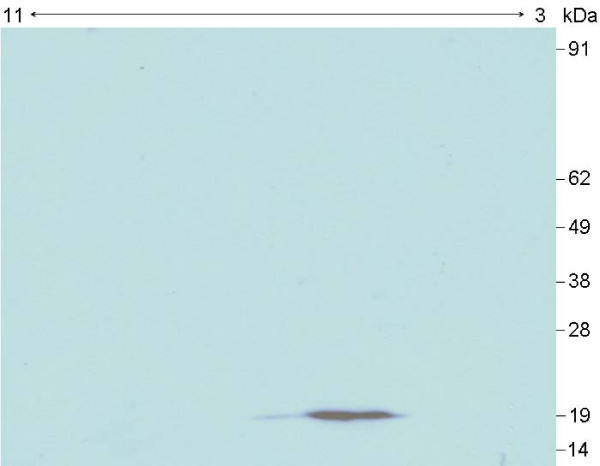
**Example of 2D ALBI assay**. Two-dimensional (pH 3-11NL) immunoblot of a whole cell sonicate of *B. garinii *serotype 4 (strain PBi) against human serum, showing a reactive protein of ~19 kDa.

Both *B. afzelii *and *B. burgdorferi *s.s. ~26 kDa proteins bound human fH (Figure 2, panels A and B, lanes 1 and 2, respectively). A ~19 kDa protein of *B. garinii *serotype 4 and a ~17 kDa protein of *B. garinii *serotype 7 showed human fH binding ability (Figure 2, panels A and B, lanes 4 and 7, respectively; Figure 3), while other *B. garinii *serotypes (3, 6, and 8) did not express any human fHBP (Figure 2, panels A and B, lanes 3, 6 and 8, respectively). Also, a ~26 kDa fHBPs was observed in *B. garinii *serotype 5 when using purified human fH (Figure 2, Panel A, lane 5), although the corresponding band when using human serum (Figure 2, Panel B, lane 5) was not observed, probably due to the lesser amount of fH present in human serum compared to the amount of purified fH used in the experiment. *B. valaisiana, B. andersonii, B. bissettii *and *B. japonica *also expressed human fHBPs (Figure 2, panels A and B, lanes 9, 10, 12 and 13, respectively), although *B. bissettii *binding was weaker when using human serum than purified human factor H, again probably due to the lesser amount of fH present in human serum. Conversely, *B. lusitaniae *did not show any human fHBP in any of the assays (Figure 2, panels A and B, lane 11). *B. parkeri *expressed a ~23 kDa human fHBP in our study (Table 1; Figure 2, panels A and B, lane 15). A ~20 kDa human fH putative ligand of *B. hermsii *was also observed in our study (Table 1; Figure 2, panels A and B, lane 14). Consistent with earlier report [3], in the case of *B. anserina*, the causative agent of avian borreliosis, and *B. coriaceae*, the causative agent of epizootic bovine abortion, no human fHBPs were observed.

### Animal fHBPs were also observed in different Borrelia species

Some of the murine fH putative ligands that showed no affinity to human fH in the study were ~15 kDa protein of *B. afzelii*, ~28 kDa protein of *B. garinii *serotype 4, ~28 and ~40 kDa proteins of *B. bissettii*, ~15 and ~26 kDa proteins of *B. japonica*, and ~40 and ~58 kDa proteins of *B. coriaceae *(Table 1; Figure 2, panel C, lanes 1, 4, 12, 13 and 17, respectively).

Amongst Lyme disease *Borreliae*, only *B. japonica *~15 kDa, ~19 kDa and ~24 kDa proteins showed rat fH binding (Table 1), while no affinity for guinea pig fH was observed. However, *B. hermsii *~20 kDa protein showed affinity for both rat and guinea pig fH (Table 1). As with mouse fH, *B. coriaceae *~58 kDa protein showed affinity for rat but not for guinea pig fH (Table 1). As expected, none of the *B. anserina *proteins showed affinity for rodent fH.

Four Lyme disease- (*B. afzelii, B. valaisiana, B. andersonii *and *B. japonica*) and none of the relapsing fever *Borrelia *expressed canine fH binding proteins (Table 1). The feline fH binding pattern in the array of *Borrelia *studied herein was similar to that of canine fH, except in VS116 (*B. valaisiana*) and 21123 (*B. andersonii*) strains, which were negative.

Finally, none of the *Borrelia *species tested, except *B. coriaceae *(~40 and ~58 kDa proteins), bound bovine fH (Table 1). Likewise, none of the *Borrelia *species tested possessed fHBPs that bind horse fH.

### Identification of human and animal fHBPs

**Figure 4:**
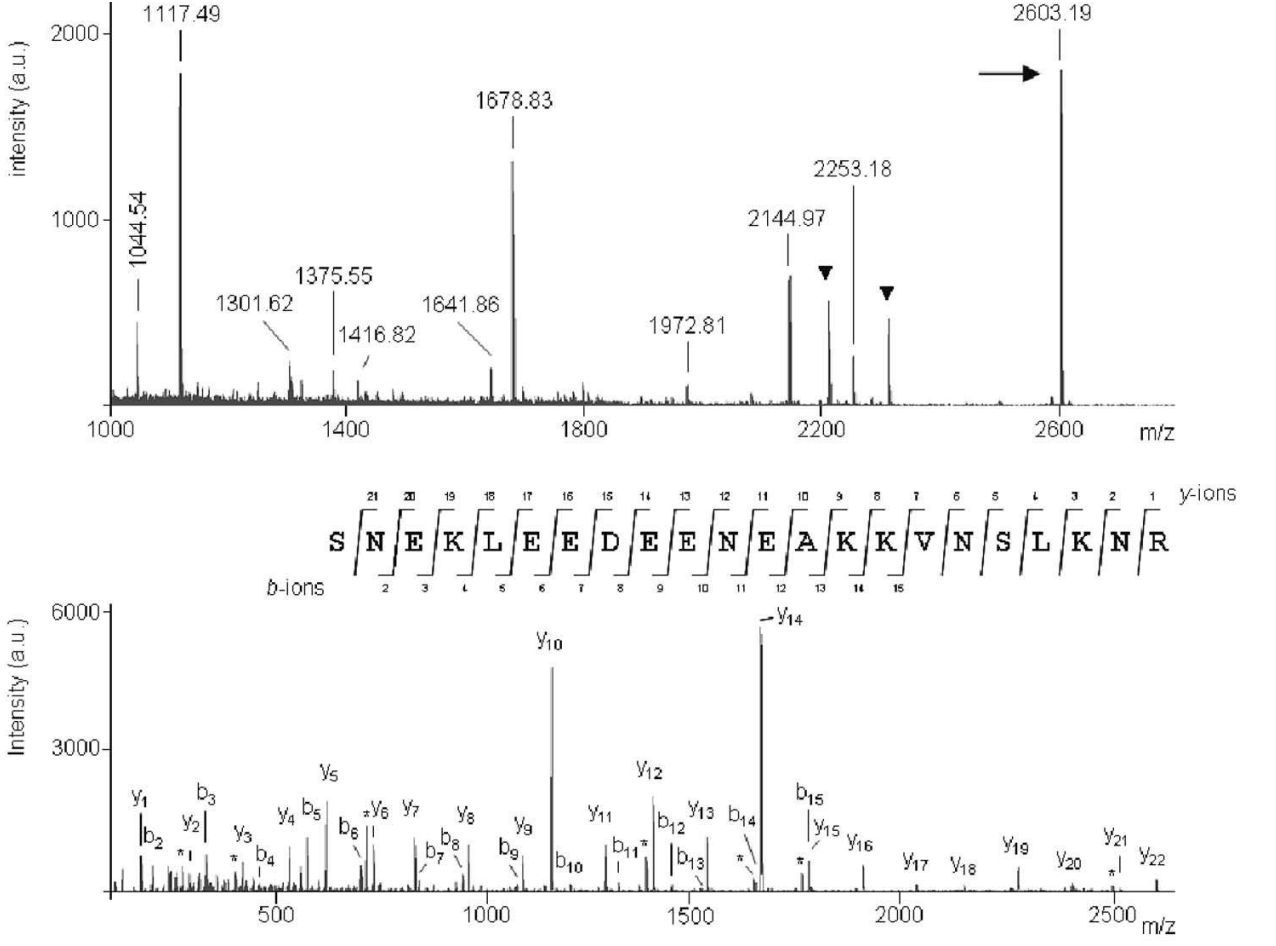
**MALDI-TOF based identification and *de-novo *sequencing**. (Top) MALDI-MS spectrum from the ~19 kDa protein of *B. garinii *ST4 (strain PBi). Relevant mass signals employed for database searching have been labeled and known trypsin and keratin peptide signals have been marked with a black triangle. The precursor ion selected for subsequent MS/MS measurement is indicated by an arrow. (Bottom) MALDI-MS/MS spectrum from the above precursor ion at m/z = 2603.19. Ions ascribed to the main fragmentation series, *y *(C-terminal series) and *b *(N-terminal series) are labeled, and C-terminal fragment ions produced by the loss of ammonia (-17 amu) are indicated by an asterisk (*). The amino acid sequence obtained by manual *de novo *sequencing is displayed showing assignment of *y *and *b *fragment ions. The letters K and L are used to indicate ambiguous ascription to glutamine/lysine and isoleucine/leucine pairs, respectively.

From the ALBI assays, we were able to identify some human fHBPs, not yet described as part of the complement evasion system of *Borrelia*. Although Mascot software failed to find any significant protein hit for the ~19 kDa protein of *B. garinii *serotype 4 (Table 1, Figure 3), manual *de novo *sequencing generated the peptide sequence SNEKLEEDEENEAQQVNSLQNR (Figure 4). The short input BLAST search showed a complete sequence homology with a hypothetical protein of *B. garinii *PBi (Genbank AAU07257). Unfortunately, neither information regarding the function and topology of this hypothetical protein was available in the protein databases nor proofs of binding were provided to exclude the probability of a contamination, although *in silico *analysis indicated that there was a high probability of two coiled-coil formations near the C-terminus (120 to 147 and 118 to 152 residues with a probability of 1.00). Although coiled-coil motifs are not specific of fHBPs, their presence has been described to be required for the formation of the fH binding site [14], therefore supporting the role of this novel protein in human fH binding [14,15].

*B. japonica *~22 kDa human fHBP (Table 1) was identified as OspE-related lipoprotein (GenBank-accession AAC62921), while ~26 kDa human fH binding proteins of *B. burgdorferi *s.s. and *B. afzelii *(Table 1) were identifed as CspA (*cspA *gene, BBA68) and BaCRASP-1 (ortholog of CspA; *mmsa*71 gene) respectively.

The ~15 kDa murine fH binding *B. afzelii *protein (Table 1) was identified as an outer membrane protein [GenBank-accession YP_853823], whilst rat fH binding ~19 kDa protein of *B. japonica *(Table 1) was identified as OspE-related lipoprotein (GenBank-accession AAC62921). Both proteins showed acidic pIs (4.0 – 5.2) in 2-DE in agreement with their theoretical pI (data not shown). The Mascot search for mouse fH-binding ~28 kDa protein of *B. garinii *serotype 4 showed a significant match and pI in agreement with the recently described BgCRASP-1 (likely to be orthologous of CspA; GenBank-accession CAH10086).

We identified a ~58 kDa *B. coriaceae *protein (Table 1) as a member of the bacterial extracellular solute-binding protein (BESBP) family. In fact, this is a probable lipoprotein according to the known features for lipoprotein signal peptides in spirochaetes [17]. For the ~40 kDa bovine fHBP of this species (Table 1) no confident protein match was found. Manual *de novo *sequencing for ~20 kDa *B. hermsii *protein (Table 1) using the corresponding MS/MS fragmentation spectra yielded two putative sequences: TLDNLLK (815.9 Da) and YLLVIFLLLSLASCDLFLK (2185.2 Da), which revealed high homology with the earlier characterized FhbA [18] of *B-hermsii*. (GenBank-accession AAY42861).

## Discussion

Among the putative human fHBPs, we have found a ~19 kDa *B. garinii *serotype 4 protein different (only 12.3% of amino acid sequence similarity) from the earlier reported BgCRASP-1 described in this genospecies [19], as well as from the earlier cited *B. burgdorferi *s.s. or *B. afzelii *fHBPs. It is proposed that the fHBPs of Lyme disease borreliae possess linear sequence elements involved in fH binding [20]. However, a sequence similarity of 15 to 18% between CspA and the rest of Erp related fHBPs (ErpA, ErpC and ErpP) indicates that this may not always be the case. A further detailed study has provided evidence that fH binding is rather dependant on protein conformation [5], and formation of coiled-coil motifs [10,14]. The high probability of coiled-coil formation observed in *in silico *analysis for this putative ~19 kDa human fHBP and its amino acid divergence from other fHBPs strengthens these findings.

*B. garinii *is the most heterogeneous species in terms of plasmid content and OspA serotype [21,22], and these differences could account for different fHBP expression and complement susceptibility. Therefore, it is not at all surprising that some of the *B. garinii *serotypes that bound human fH in this study (OspA serotypes 4 and 7) also resisted complement mediated killing in a previous experiment [7]. Human fH binding by *B. valaisiana *could correspond to its proposed ability to produce erythema migrans [23], and the absence of human fH binding by *B. anserina *and *B. coriaceae *observed in our experiments correlates with the fact that these species do not infect humans.

The host selectivity of different *Borrelia *species correlates with their complement sensitivity, and thus also with their fH binding profile [7,24,25]. We show novel murine fH putative ligands expressed by Lyme disease *Borrelia *and *B. coriaceae*. The murine fH binding ability of *B. bissettii *and *B. japonica *strengthens their survival in rodents [26,27]. Moreover, the putative murine fHBP identified herein in *B. garinii *OspA serotype 4 correlates with the described ability of these strains to survive in mice [28].

We and others have reported that cattle and horses are not suitable hosts for Lyme disease related *Borreliae *[7,24,29], which strongly correlates with the inability of borrelial proteins to bind bovine and equine fH. However, binding of bovine fH only by *B. coriaceae *(~40 kDa & ~58 kDa proteins) suggests that cattle are a primary host for this *Borrelia *species. We have found a ~58 kDa protein that appears to have a bovine fH binding ability, to be a BESBP that may be an immune evasion tool of *B. coriaceae *in bovine. Like in other fHBPs, the ~58 kDa protein is a putative lipoprotein, as per the requirements described by Setubal *et al*. [17]., which suggest that it is located in the membrane.

The role of carnivores as hosts for *Borrelia *is probably limited. In this study we have noticed canine fHBPs in *B. afzelii *and *B. valaisiana *and, although with weak signals, also in *B. andersoni *and *B. japonica*, that could account for the ability of these genospecies to infect dogs [30]. Feline fHBPs has been detected, as well, in *B. afzelii *and *B. japonica*, although neither good data regarding reservoir competence nor pathogenicity of *Borrelia *in these hosts is available.

In summary, the fH binding strategy employed by *Borrelia *species in different hosts is complex. Novel, putative human and animal fHBPs have been found in this study, which highlights the multiplicity of the immune evading arsenal that *Borrelia *possesses.

## Competing interests

The authors declare that they have no competing interests.

## Authors' contributions

MRB carried out the affinity ligand binding assays, the 2-DE electrophoresis and drafted the manuscript. RE participated in the design of the study and carried out the preparation of protein lysates of the *Borrelia *species tested. EC performed the identification of protein spots. HG and IJ also participated in the design of the study and helped in the 2-DE electrophoresis. PA conceived of the study, and participated in its design and coordination and helped to draft the manuscript. All authors read and approved the final manuscript.
